# Comparison of the Changes in Visceral Adipose Tissue After Lobectomy and Segmentectomy for Patients With Early‐Stage Lung Cancer

**DOI:** 10.1002/jcsm.13751

**Published:** 2025-03-04

**Authors:** Tetsuya Isaka, Takuya Nagashima, Kota Washimi, Haruhiro Saito, Hiroto Narimatsu, Shunsuke Shigefuku, Chiaki Kanno, Ryotaro Matsuyama, Naoko Shigeta, Yui Sueishi, Hiroyuki Ito

**Affiliations:** ^1^ Department of Thoracic Surgery Kanagawa Cancer Center Yokohama Kanagawa Japan; ^2^ Department of Pathology Kanagawa Cancer Center Yokohama Kanagawa Japan; ^3^ Department of Thoracic Oncology Kanagawa Cancer Center Yokohama Kanagawa Japan; ^4^ Department of Genetic Medicine Kanagawa Cancer Center Yokohama Kanagawa Japan; ^5^ Cancer Prevention and Cancer Control Division Kanagawa Cancer Center Research Institute Yokohama Kanagawa Japan; ^6^ Graduate School of Health Innovation Kanagawa University of Human Services Kawasaki Kanagawa Japan

**Keywords:** early‐stage lung cancer, less invasive, lobectomy, segmentectomy, visceral fat, waist circumference

## Abstract

**Background:**

The impact of lobectomy versus segmentectomy on body composition changes, particularly adipose tissue, in patients with early‐stage lung cancer remains unclear. This study aimed to determine the association between these surgical approaches and postoperative changes in adipose tissue.

**Methods:**

We retrospectively analysed visceral fat area (VFA) and waist circumference (WC) at the L3 level using cross‐sectional computed tomography images from 346 recurrence‐free patients who underwent lobectomy (*n* = 240) or segmentectomy (*n* = 106) for clinical stage 0–I primary lung cancer between January 2016 and December 2018. Long‐term postoperative changes in VFA and WC by the third postoperative year (POY3) were compared between the lobectomy and segmentectomy groups using two‐way repeated measures analysis of variance (ANOVA). Risk factors for VFA reduction were identified through multivariable analysis using logistic regression model. Propensity score matching (PSM, 1:1 matching) was also performed to compare VFA and WC changes between the lobectomy and segmentectomy groups.

**Results:**

At 6 months postoperatively, VFA and WC decreased by 16.4% and 1.0% in the lobectomy groups, respectively, and increased by 0.1% and 0.2% in the segmentectomy groups (*p* < 0.001 and *p* = 0.029, respectively). The two‐way repeated measure ANOVA showed that the VFA and WC significantly decreased in the lobectomy group compared with the segmentectomy group within the POY3 (*p* < 0.001 and *p* = 0.038, respectively). Patients with a VFA change of ≥ −13% at POY3 (*n* = 238) had significantly better OS than those with a change of < −13% (*n* = 108) (5‐year OS rate, 97.7% vs. 93.4%, *p* = 0.017), and VFA change < −13% at POY3 was an independent poor prognostic factor for OS (hazard ratio, 4.14; *p* = 0.013). Lobectomy was identified as an independent risk factor for a VFA change of < −13% at POY3 (odds ratio, 2.86; *p* < 0.001). After PSM (*n* = 93 for each group), VFA and WC significantly decreased in the lobectomy group compared with the lobectomy group within the POY3 (*p* = 0.009 and *p* = 0.020, respectively).

**Conclusions:**

In patients with early‐stage lung cancer without recurrence, long‐term postoperative changes in VFA and WC differed between lobectomy and segmentectomy. Lobectomy resulted in a greater decrease in VFA from 6 months to 3 years postoperatively. In contrast, segmentectomy was associated with neither long‐term postoperative VFA nor WC reduction.

**Abbreviations**
ANOVA: analysis of variance
BMI: body mass index
CI: confidence interval
CT: computed tomography
FEV1: forced expiratory volume in 1 s
HR: hazard ratio
OR: odds ratio
OS: overall survival
POY: postoperative year
PSM: propensity score matching
VFA: visceral fat area
WC: waist circumference

## 1 Introduction

Although lobectomy is the standard procedure for early‐stage primary lung cancer [1], recent clinical trials have demonstrated that segmentectomy is an acceptable alternative procedure [2, 3, 4]. In the JCOG0802/WJOG4607L clinical trial, the overall survival (OS) was compared after segmentectomy and lobectomy for early‐stage non‐small cell lung cancer with solid‐dominant tumours of ≤ 2 cm in size. Segmentectomy was associated with a significantly better OS than lobectomy (5‐year OS rate, 94.3% vs. 91.1%; *p* < 0.0001) [2]. Furthermore, the non–lung cancer‐related deaths were less frequent after segmentectomy than after lobectomy [2]. This may account for the difference in OS between the two procedures and highlights the minimally invasive nature of segmentectomy.

Recent clinical trials and meta‐analyses have reported that segmentectomy preserves respiratory function better than lobectomy within the first postoperative year [2, 3, 5]. However, long‐term follow‐up demonstrates that the difference in respiratory function between the two surgical techniques diminishes over time [6, 7]. Further studies are necessary to elucidate the physiological changes that may influence the prognosis of patients undergoing lobectomy or segmentectomy.

We have previously demonstrated that reduction of psoas major muscle mass persists after lobectomy, not segmentectomy, in patients with early‐stage lung cancer [8]. The decrease in psoas major muscle mass after segmentectomy was 4.7% less than that after lobectomy at the 3‐year postoperative follow‐up [8]. This indicates that segmentectomy may prevent the progression to sarcopenia and preserve the patient's body and function long after surgery.

In this study, we focused on the changes in adipose tissue after segmentectomy and lobectomy. According to recent studies, adipose tissue, including visceral fat and subcutaneous fat, are associated with the prognosis of patients with primary lung cancer [9, 10, 11, 12, 13, 14]. Visceral fat and waist circumference (WC) represent the true visceral obesity or body fat mass, which affects the patient's immune system, inflammatory status and nutrition [14, 15, 16, 17, 18]. Several recent studies have demonstrated that a low visceral fat content, or adipopenia, is associated with poor prognosis in patients with lung cancer [12, 13, 14]. Patients with lung cancer and adipopenia have reduced energy stores, which induces systemic inflammation following tumour or surgical intervention and increases the mortality risk, especially of non‐cancer‐related death, due to energy imbalance [9, 19, 20].

Maintenance of body composition, including visceral fat and WC, is important following minimally invasive surgery for lung cancer. However, the differences in body composition changes between lobectomy and segmentectomy for early‐stage lung cancer remain insufficiently understood. Thus, we aimed to compare the amount of adiposity change after segmentectomy and lobectomy and determine its association with prognosis in patients with lung cancer.

## 2 Material and Methods

### 2.1 Patients and Surgical Procedures

The study was approved by the review board of the Kanagawa Cancer Center (No: 2023 Eki‐32; 12 June 2023). The need for obtaining consent was waived due to the retrospective nature of the study.

**FIGURE 1:**
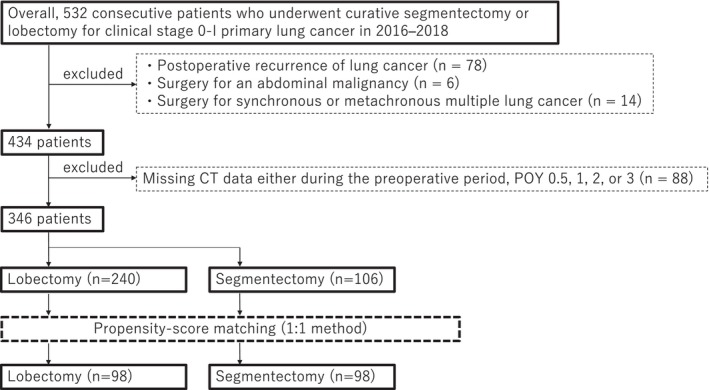
Consort diagram of this study. POY, postoperative year.

Among 532 consecutive patients who underwent lobectomy or segmentectomy for clinical stage 0–I primary lung cancer (8th edition TNM) at the Kanagawa Cancer Center from January 2016 to December 2018, 78 experienced lung cancer recurrence postoperatively, 14 underwent surgery for synchronous or metachronous multiple lung cancers and 6 had surgery for an abdominal malignancy or required long‐term dietary restrictions due to its complications, thereby excluding them from the study (Figure 1). From the remaining 434 patients, 346 (lobectomy, *n* = 240; segmentectomy, *n* = 106) patients undergoing abdominal and chest computed tomography (CT) preoperatively and postoperatively every 6–12 months (postoperative year [POY] 0.5), 12–24 months (POY1), 24–36 months (POY2) and 36–48 months (POY3) were included in the study (Figure 1).

The segmentectomy group included patients who underwent segmentectomy with adjacent subsegmentectomy. This study included patients with early‐stage lung cancer who received intentional segmentectomy and passive segmentectomy in the segmentectomy group. Intentional segmentectomy was performed for patients with small‐sized (≤ 2 cm in size) or ground‐glass opacity‐predominant tumours. Passive segmentectomy was performed for patients with solid‐predominant tumours greater than 2 cm, as they were intolerable for lobectomy because of the patient's advanced age, low lung function or comorbidities. Lobectomy was performed for patients with early‐stage lung cancer who had small‐sized or ground‐glass opacity‐predominant tumours of ≤ 2 cm in diameter that are difficult to resect by segmentectomy with adequate margins or patients who had solid‐predominant tumours of > 2 cm in diameter and could tolerate lobectomy. The patient's procedure was determined at a surgical conference based on patients' health condition and radiographic findings of the tumour.

### 2.2 Follow‐Up

Chest and abdominal CT were performed postoperatively every 6–12 m for 5 years. Additionally, blood tumour markers were evaluated postoperatively every 3–6 m for the first 3 years and thereafter every 6–12 m up to 5 years. If recurrence was suspected, positron emission tomography–CT or magnetic resonance imaging of the head was also performed. Patients without recurrence of lung cancer during the follow‐up were eligible for this study.

### 2.3 Measurements and Calculation of Visceral Fat Areas and Waist Circumference Changes

CT scans were conducted using an Aquillion CT scanner (Toshiba Medical Systems, Tochigi, Japan), specifically the model AquilionCXL (120 kV, 600 mA), AquilionONE (120 kV, 580 mA) or AquilionRX (120 kV, 500 mA). Visceral fat area (VFA) and WC were measured using unenhanced axial CT images at the L3 level [9, 12, 13, 16, 21, 22] with a 5‐mm section thickness. These measurements were automatically calculated using SYNAPSE VINCENT (Fujifilm Medical Co., Ltd., Tokyo, Japan) by T.I., who was blinded to the patients' clinical and pathological data (Figure S1). VFA was quantified automatically with thresholds ranging from −200 to −50 Hounsfield units. Preoperative CT was obtained within 3 months before surgery. The amount of change in postoperative VFA and WC from the perioperative period was calculated as follows:

$$\begin{array}{c} \text{Percentage of change}\;\left(\%\right)=\\ \frac{\left(\text{postoperative value}\;\right)-\left(\text{preoperative value}\right)}{\text{preoperative value}\;}\times 100. \end{array}$$

### 2.4 Statistical Analyses

The primary objective of this study is to compare the long‐term VFA and WC change (up to 3 years) after lobectomy versus segmentectomy. The secondary objective is to analyse whether long‐term changes in VFA and WC are related to the surgical procedure and OS.

Mann–Whitney *U* or Student's *t* test and Fisher's exact test were used to compare continuous and categorical variables, respectively, between the study groups. VFA and WC changes at each postoperative period (POY0.5, POY1, POY2 and POY3) from the preoperative period were analysed using one‐way repeated measures analysis of variance (ANOVA) and Bonferroni adjustment. Two‐way repeated measure ANOVA was used to compare the VFA and WC changes between the study groups.

The optimal cutoff values for VFA and WC changes at POY3 were examined for survival analysis based on a Cox proportional hazard model, and the most significant threshold was used throughout all further analyses. OS was defined as the period from the date of surgery to the date of all‐cause death, wherein patients were censored without any event in the last observation period. OS for the small and large VFA and WC reduction groups at POY3 was analysed using the Kaplan–Meier method and compared with the log‐rank test. A Cox proportional hazard regression model was used for univariable and multivariable (stepwise method) analyses for OS.

The risk factor for the VFA and WC change at POY3 was assessed using univariable and multivariable logistic regression analysis (the multivariable analysis was performed on all variables with a *p* value of < 0.10 in the univariate analysis). Following variables are included in the univariable and multivariable analysis: age, sex, body mass index (BMI), smoking history, Charlson comorbidity index, surgical approach (complete video‐assisted thoracoscopic surgery or open thoracotomy), postoperative complications, histology, tumour size, pathological stage, adjuvant chemotherapy and extent of lung resection (segmentectomy or lobectomy).

To reduce selection bias between patients who underwent lobectomy and segmentectomy, propensity score matching (PSM) was performed (1:1 matching, calliper = 0.01) using the following preoperative factors: age, sex, BMI, smoking history, Charlson comorbidity index, surgical approach, histology, tumour size, pathological stage and adjuvant chemotherapy. After PSM, the VFA and WC change between segmentectomy and lobectomy were compared using two‐way repeated measures ANOVA.

Statistical significance was set at *p* < 0.05. All statistical analyses were performed using EZR on R Commander (version 1.30; Saitama Medical Center, Jichi Medical University, Saitama, Japan), a graphical user interface for R (The R Foundation for Statistical Computing, Vienna, Austria).

## 3 Results

The median follow‐up period was 5.2 years (5.0–5.9 years). As shown in Table 1, the maximum tumour size was larger, and the pathologic stage was higher in the lobectomy group than in the segmentectomy group. Postoperative adjuvant chemotherapy was administered more frequently in the lobectomy group than in the segmentectomy group. There was no significant difference in other characteristics between the study group. Of the 106 patients who underwent segmentectomy, 87 patients (82.1%) received intentional segmentectomy, whereas only 19 patients (17.9%) received passive segmentectomy.

**TABLE 1:** Comparison of characteristics of patients who underwent lobectomy versus segmentectomy for clinical stage 0–I primary lung cancer.

Total *n* = 346		Segmentectomy (*n* = 106)	Lobectomy (*n* = 240)	*p* values^a^
Age, median (IQR), years		69.5 (62.3–74.0)	69.0 (64.0–75.0)	0.628^b^
Male, No. (%)		46 (43.6)	119 (49.6)	0.296
Height, median (IQR), cm		159.6 (153.0–164.5)	160.4 (152.1–166.8)	0.523^b^
Weight, median (IQR), kg		58.8 (50.0–63.2)	56.7 (49.2–64.3)	0.560^b^
Body mass index, median (IQR), kg/m^2^		22.8 (20.6–25.0)	22.2 (20.5–24.4)	0.181^b^
Smoking history, No. (%)		65 (61.3)	125 (52.1)	0.128
FEV1%, median (IQR), %		74.6 (70.6–79.4)	74.1 (69.0–79.1)	0.277^b^
%VC, mean (SD), %		108.7 (15.0)	109.6 (14.5)	0.613^c^
Other cancer history, No. (%)		27 (25.5)	53 (22.1)	0.492
Comorbidity, No. (%)				
Chronic obstructive pulmonary disease		5 (4.7)	14 (5.8)	0.801
Coronary disease		7 (6.6)	7 (2.9)	0.138
Hypertension		40 (37.7)	83 (34.6)	0.626
Diabetes mellitus		19 (17.9)	27 (11.2)	0.121
Charlson comorbidity index ≥ 1, No. (%)		54 (50.9)	113 (47.1)	0.560
cVATS, No. (%)		43 (40.6)	87 (36.2)	0.471
Postoperative complications ≥ G2^d^, No. (%)		24 (22.6)	59 (24.6)	0.785
Adenocarcinoma, No. (%)		98 (92.5)	214 (89.2)	0.435
Maximum tumour size ≥ 2.0 cm, No. (%)		27 (25.5)	151 (62.9)	< 0.001
Pathological stage 0–I, No. (%)		105 (99.1)	212 (88.3)	< 0.001
Adjuvant chemotherapy, No. (%)		0 (0)	42 (17.5)	< 0.001
Preoperative VFA, mean (SD), cm^2^		110.9 (74.9)	114.0 (78.1)	0.726^c^
Preoperative WC, mean (SD), cm		83.2 (10.7)	82.9 (10.3)	0.820^c^
Preoperative SFA, mean (SD), cm^2^		108.5 (53.7)	101.9 (51.6)	0.278^c^
VFA change	POY0.5, mean (SD), %	0.1 (39.4)	−16.4 (26.1)	< 0.001^c^
	POY1, mean (SD), %	5.0 (45.7)	−11.3 (33.8)	< 0.001^c^
	POY2, mean (SD), %	7.5 (54.9)	−9.3 (33.8)	< 0.001^c^
	POY3, mean (SD), %	11.6 (43.9)	−6.7 (37.4)	< 0.001^c^
WC change	POY0.5, mean (SD), %	0.2 (4.7)	−1.0 (4.6)	0.029^c^
	POY1, mean (SD), %	0.6 (5.0)	−0.3 (4.9)	0.111^c^
	POY2, mean (SD), %	1.0 (5.5)	−0.3 (5.0)	0.031^c^
	POY3, mean (SD), %	1.5 (4.9)	−0.1 (5.9)	0.015^c^
SFA change	POY0.5, mean (SD), %	5.0 (20.5)	−0.3 (28.2)	0.083^c^
	POY1, mean (SD), %	6.8 (24.7)	3.7 (34.2)	0.396^c^
	POY2, mean (SD), %	7.4 (28.2)	3.8 (36.9)	0.369^c^
	POY3, mean (SD), %	8.9 (23.6)	4.5 (36.1)	0.241^c^

Abbreviations: %VC, percentage predicted vital capacity; cVATS, complete video‐assisted thoracoscopic surgery; FEV1%, percentage predicted forced expiratory volume at 1 s; IQR, interquartile range; POY, postoperative year; SD, standard deviation; SFA, subcutaneous fat area; VFA, visceral fat area; WC, waist circumference.

^a^ Fisher's exact test.

^b^ Mann–Whitney U test.

^c^ Student *t* test.

^d^ Based on Clavien–Dindo classification.

The VFA significantly decreased in the lobectomy group compared with the segmentectomy group at each postoperative period (*p* < 0.001, respectively; Table 1). The WC significantly decreased at POY0.5 (*p* = 0.029), POY2 (*p* = 0.031) and POY3 (*p* = 0.015) in the lobectomy group compared with the segmentectomy group (Table 1). The histogram of VFA and WC change (%) at POY0.5 and POY3 was shown in Figure S2.

**FIGURE 2:**
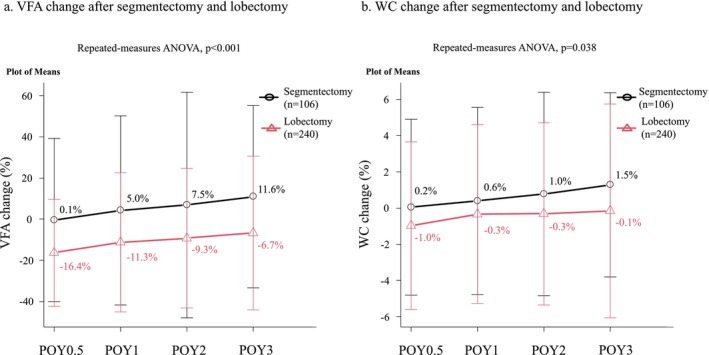
Comparison of VFA and WC changes after segmentectomy and lobectomy. The (a) VFA and (b) WC were considerably decreased in the lobectomy group within the POY3 compared with the segmentectomy group. ANOVA, analysis of variance; POY, postoperative year; VFA, visceral fat area; WC, waist circumference.

**FIGURE 3:**
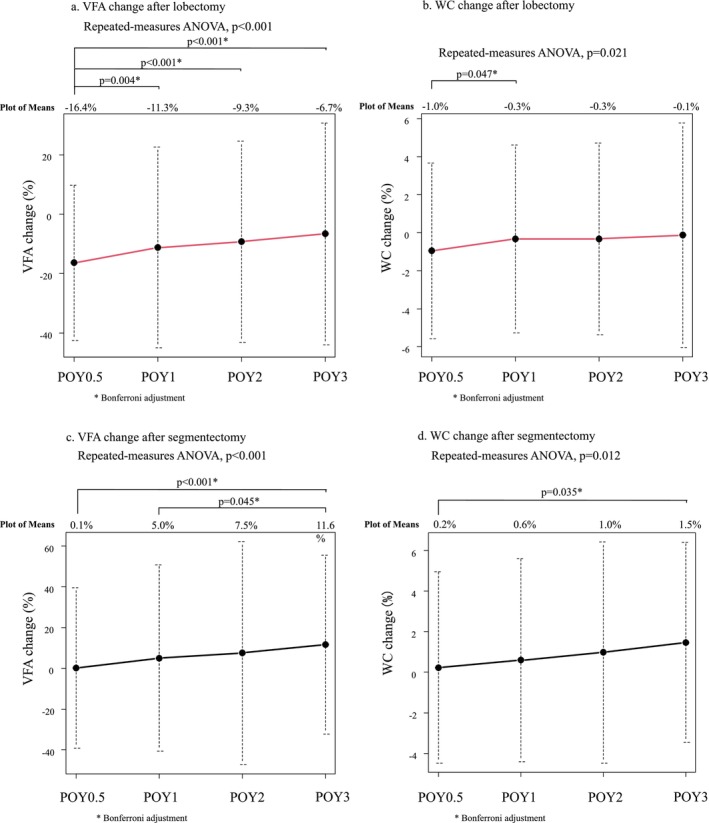
Postoperative changes in VFA and WC after lobectomy and segmentectomy. (a, b) The VFA and WC decreased at POY0.5 and gradually increased over time after lobectomy. (c, d) The VFA and WC did not decrease over the postoperative period in the segmentectomy group. ANOVA, analysis of variance; POY, postoperative year; VFA, visceral fat area; WC, waist circumference.

Two‐way repeated measures ANOVA revealed that the VFA and WC were significantly decreased in the lobectomy group within the POY3 compared with the segmentectomy group (*p* < 0.001 and *p* = 0.038, respectively; Figure 2). In the lobectomy group, VFA decreased by 16.4% at POY0.5; thereafter, the reduction in VFA became smaller (Figure 3a). WC decreased by 1.0% at POY0.5; the reduction in WC became smaller at POY1 (Figure 3b). In the segmentectomy group, the VFA and WC did not decrease over the postoperative period in the segmentectomy group (Figure 3c,d). In subgroup analysis, VFA and WC were significantly decreased in the lobectomy group within POY3 than in the segmentectomy group among the patients with ≥65 years of age (*p* < 0.001 and *p* = 0.006, respectively; Figure S3a,b). However, VFA and WC changes between the lobectomy and segmentectomy groups were similar among patients < 65 years of age (*p* = 0.095 and *p* = 0.850, respectively; Figure S3c,d).

**FIGURE 4:**
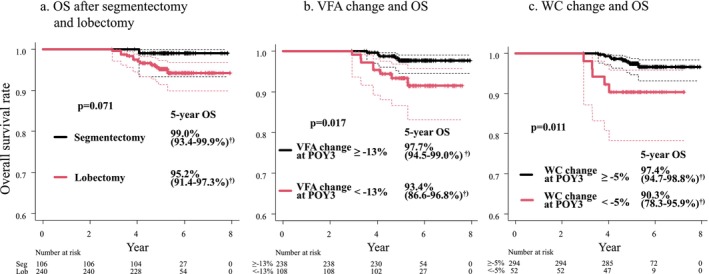
OS after segmentectomy and lobectomy. (a) OS tended to be better in the segmentectomy group than in the lobectomy group. (b) OS of patients with a VFA change of ≥ −13% at POY3 was significantly better than that of patients with a VFA change of < −13% at POY3. (c) OS of patients with a WC change of ≥ −5% at POY3 was considerably better than that of patients with a WC change of < −5% at POY3. ^†^95% CI. CI, confidence interval; OS, overall survival; POY, postoperative year; VFA, visceral fat area; WC, waist circumference.

Of the 13 deaths in the study, 12 occurred in the lobectomy group. The OS tended to be better in the segmentectomy group than in the lobectomy group (5‐year OS rate: 99.0% vs. 95.2%, *p* = 0.071; Figure 4a). The cut‐off value for VFA and WC changes at POY3 was to be −13% (hazard ratio [HR], 3.58; 95% confidence interval [CI], 1.17–10.94; *p* = 0.025) and −5% (HR, 3.82; 95% CI: 1.25–11.7; *p* = 0.019) (Figure S4). Patients with a VFA change of ≥ −13% at POY3 had significantly better OS compared to those with a VFA change of < −13% at POY3 (5‐year OS rate: 97.7% vs. 93.4%, *p* = 0.017; Figure 4b). Similarly, patients with a WC change of ≥ −5% at POY3 had significantly better OS than those with a WC change of <−5% at POY3 (5‐year OS rate: 97.4% vs. 90.3, *p* = 0.011; Figure 4c). In a multivariable analysis of OS, VFA change < −13% at POY3 was a significant poor prognostic factor (HR, 4.14; 95% CI, 1.35–12.7; *p* = 0.013) (Table 2).

**TABLE 2:** Univariable and multivariable (stepwise method) analysis for overall survival using Cox proportional hazards regression model.

	Univariable analysis			Multivariable analysis		
Variable	HR	95% CI	*p* values	HR	95% CI	*p* values
Age ≥ 65	2.19	0.49–9.88	0.308			
Male	3.84	1.06–14.0	0.041	4.44	1.22–16.2	0.024
BMI ≤ 18.5 kg/m^2^	0.92	0.12–7.12	0.940			
Smoking history	1.88	0.58–6.12	0.292			
CCI ≥ 1	1.29	0.43–3.85	0.645			
Lobectomy (vs. segmentectomy)	5.33	0.69–41.0	0.108			
Open thoracotomy (vs. cVATS)	0.94	0.31–2.86	0.906			
Postoperative complications ≥ G2	0.25	0.03–1.96	0.189			
Nonadenocarcinoma	3.02	0.83–11.0	0.093			
Tumour size ≥ 2.0 cm	3.21	0.88–11.7	0.076			
Pathological stage 0–I (vs. ≥ II)	3.73	1.03–13.6	0.046			
Adjuvant chemotherapy	0.59	0.08–4.51	0.609			
WC change < −5% at POY3	3.82	1.25–11.7	0.019			
VFA change < −13% at POY3	3.58	1.17–10.9	0.025	4.14	1.35–12.7	0.013

Abbreviations: BMI, body mass index; CCI, Charlson comorbidity index; CI, confidence interval; cVATS, complete video‐assisted thoracoscopic surgery; OR, odds ratio; POY, postoperative year; VFA, visceral fat area; WC, waist circumference.

In the multivariable analysis, lobectomy was an independent risk factor for VFA change < −13% (OR, 2.90; 95% CI, 1.62–5.20; *p* < 0.001) and for WC change < −5% at POY3 (OR, 4.30; 95% CI, 1.60–11.6; *p* = 0.004) (Table 3).

**TABLE 3:** Univariable and multivariable analysis of risk factors for VFA and WC reduction at POY3 using Logistic regression model.

	VFA change < −13% at POY3				WC change < −5% at POY3			
Variable	Univariable analysis		Multivariable analysis		Univariable analysis		Multivariable analysis	
	OR (95% CI)	*p* values	OR (95% CI)	*p* values	OR (95% CI)	*p* values	OR (95% CI)	*p* values
Age ≥ 65	1.73 (1.00–2.98)	0.048	1.73 (0.98–3.05)	0.057	1.49 (0.73–3.04)	0.272		
Male	0.67 (0.42–1.05)	0.082	0.76 (0.42–1.38)	0.367	1.02 (0.56–1.84)	0.951		
BMI ≤ 18.5 kg/m^2^	1.73 (0.79–3.81)	0.170			0.94 (0.31–2.82)	0.909		
Smoking history	0.51 (0.32–0.81)	0.004	0.65 (0.36–1.16)	0.142	0.78 (0.44–1.43)	0.440		
CCI ≥ 1	0.89 (0.57–1.41)	0.621			1.19 (0.66–2.14)	0.567		
Lobectomy (vs. segmentectomy)	2.93 (1.66–5.19)	<0.001	2.90 (1.62–5.20)	< 0.001	4.92 (1.90–12.8)	0.001	4.30 (1.60–11.6)	0.004
Open thoracotomy (vs. cVATS)	1.10 (0.68–1.76)	0.705			1.42 (0.76–2.69)	0.273		
Postoperative complications ≥ G2^a^	1.01 (0.59–1.71)	0.980			0.53 (0.24–1.18)	0.120		
Nonadenocarcinoma	0.91 (0.42–1.98)	0.811			1.87 (0.80–4.40)	0.149		
Tumour size ≥ 2.0 cm	1.42 (0.90–2.24)	0.136			1.78 (0.97–3.28)	0.062	1.20 (0.63–2.29)	0.574
Pathological stage 0–I (vs. ≥ II)	1.62 (0.75–3.53)	0.221			2.36 (0.99–5.67)	0.054	1.69 (0.69–4.13)	0.247
Adjuvant chemotherapy	0.87 (0.43–1.77)	0.694			0.74 (0.28–1.98)	0.547		

Abbreviations: BMI, body mass index; CCI, Charlson comorbidity index; CI, confidence interval; cVATS, complete video‐assisted thoracoscopic surgery; OR, odds ratio; POY, postoperative year; VFA, visceral fat area; WC, waist circumference.

^a^ Clavien–Dindo classification.

**FIGURE 5:**
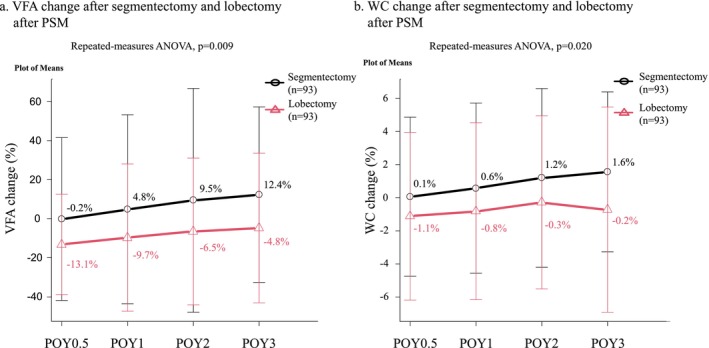
Comparison of VFA and WC changes in the segmentectomy and lobectomy groups after PSM. (a) The postoperative VFA change was significantly smaller in the segmentectomy group than in the lobectomy group. (b) The postoperative WC change was significantly smaller in the segmentectomy group than in the lobectomy group. ANOVA, analysis of variance; POY, postoperative year; PSM, propensity score matching; VFA, visceral fat area; WC, waist circumference.

After PSM, the clinicopathological features of the lobectomy and segmentectomy groups were comparable (Table S1). In addition, VFA and WC reduction were significantly greater in the lobectomy group than in the segmentectomy group within the POY3 (*p* = 0.009 and *p* = 0.020, respectively) (Figure 5a,b).

## 4 Discussion

This study is the first to demonstrate that long‐term postoperative changes in VFA and WC differ between lobectomy and segmentectomy in patients with early‐stage lung cancer without recurrence. Lobectomy was associated with a reduction in VFA and WC from POY0.5 to POY3 postoperatively, whereas segmentectomy was not associated with a reduction in VFA or WC.

Segmentectomy preserves more lung function, including FEV1 and forced vital capacity, than lobectomy [2, 3, 5, 23]. In the JCOG0802 trial, the FEV1 reduction rates at 6 m after segmentectomy and lobectomy were 10.4% and 13.1%, respectively (*p* < 0.0001) and at 12 m were 8.5% and 12.0% (*p* < 0.0001), respectively [2]. In the CALGB140503 trial, the FEV1 reduction rate at 6 m was 4.0% and 6.0% after segmentectomy and lobectomy, respectively [3]. In the JCOG0802 trial, there were significantly fewer non–lung cancer‐related deaths in the segmentectomy group than in the lobectomy group [2]. However, it was not clarified whether the minimal differences in respiratory function between the two procedures can affect the incidence of non–lung cancer‐related deaths. Hence, further studies are required to assess the differences in long‐term physiological changes in patients after segmentectomy and lobectomy.

In adipopenia, a patient's energy stores are reduced due to lipolysis and decreased adipocyte size [24]. Adipopenia is associated with poor prognosis in patients with cancer because tumours and surgery may induce systemic inflammation and further energy imbalance [9, 19, 20]. However, obesity is associated with major cardiovascular risk factors, including hypertension, dyslipidemia, type 2 diabetes mellitus, chronic inflammation, and nonalcoholic fatty liver disease [25]. In patients with cancer, obesity is associated with a better prognosis [10, 26, 27], a phenomenon known as the ‘obesity paradox’. Adipopenia is associated with poor prognosis in patients with primary lung cancer [9, 10, 11, 14], pancreatic cancer [28], diffuse large B‐cell lymphoma [21] and prostate cancer [22]. COPD also exhibits the obesity paradox [29].

Visceral fat is the best representative of body fat mass among the other indices, and it exhibits correlations with the individual's nutritional, immune and inflammatory status [14]. Although a high visceral fat mass worsens the prognosis of young disease‐free individuals, it improves the prognosis of elderly patients with cancer and other diseases [12, 30, 31]. A Chinese multicentre prospective study of 14 018 patients with cancer demonstrated that a low VFA was associated with a high mortality risk (HR: 1.33; 95% CI: 1.08–1.64; *p* = 0.007). A 10‐cm^2^ reduction in VFA is associated with a 20% increase in cancer‐related mortality risk. Furthermore, a low VFA was a risk factor of mortality in primary lung cancer (HR: 1.27; 95% CI: 1.01–1.59; *p* = 0.040) [14]. A high VFA is reportedly associated with a better postoperative prognosis in patients with lung cancer [12, 13]. Hong et al. [32] demonstrated that the risk of all‐cause mortality decreases with increasing VFA in older Asian adults (HR: 0.64; 95% CI: 0.47–0.87). Patients with low VFA smoke more frequently, consume more alcohol, have lower nutritional and immunity status [14] and have poorer lung function [33] than those with high VFA. These factors may contribute to their poor prognosis. This study demonstrated that VFA change < −13% at POY3 is a poor prognostic factor for OS in patients with early‐stage lung cancer, suggesting the importance of preventing postoperative adipopenia.

In this study, the decrease in VFA that occurred immediately after lobectomy did not occur after segmentectomy. Segmentectomy is reportedly associated with less frequent deterioration of physical and cognitive functioning, dyspnoea and fatigue at 12 m and faster recovery of dyspnoea than lobectomy [34]. Furthermore, segmentectomy avoids the long‐term reduction of psoas muscle mass and may prevents it progression to sarcopenia [8]. The mechanism by which VFA decreases as the extent of resection increases is not well understood. Exposure to a hypoxic environment has been reported to lead to appetite suppression and reduce physiological activities, such as food intake, digestion and nutrient absorption [35, 36]. Hypoxic conditions can increase energy expenditure, leading to a negative energy balance and ultimately weight loss, primarily due to a decrease in fat mass [36]. A greater extent of lung resection may expose patients to hypoxic conditions and increase energy expenditure, which can disrupt lipid metabolism, resulting in fat depletion [24]. Furthermore, the decrease in VFA may decrease the secretion of anti‐inflammatory hormones from the adipose tissue, resulting in long‐term postoperative compromise and trauma, muscle weakness and decreased respiratory function, which worsens the prognosis [31, 33, 37].

In this subgroup analysis, VFA and WC did not decrease in the long term after segmentectomy, irrespective of patients' age (Figures 2 and S3). By contrast, VFA and WC decreased in the long term after lobectomy among patients ≥ 65 years of age, with no recovery to these preoperative levels (Figure S3a,b). However, patients aged < 65 years showed no significant difference between VFA and WC change postoperatively after lobectomy; VFA and WC decreased at POY0.5 and recovered thereafter to these preoperative levels (Figure S3c,d). These findings suggest that segmentectomy is more effective than lobectomy for preventing the progression of postoperative adipopenia, especially in elderly patients. Because older patients with fat may eat better, be more sociable and have greater overall well‐being [32], they may have a better quality of life after segmentectomy than after lobectomy.

WC is an easily measurable indicator of visceral or central obesity [17], and it reflects a truer adiposity than BMI [16, 18]. In our study, a decreased WC was detected in the lobectomy group alone. This may support the hypothesis that, as with visceral adipose fat, the maintenance of appetite after surgery in the segmentectomy group maintains body composition. A previous study has reported that an increase in WC increases the risk of mortality due to primary lung cancer [38]. However, another study stated that WC is not associated with lung cancer‐related mortality [39]. Larger studies are required to clarify the association between WC and prognosis.

This study had several limitations. First, it was a single‐centre retrospective study with a small sample size. Thus, there may be a selection bias in the two groups, despite PSM analysis. This study included patients who received intentional and passive segmentectomy, wherein only 17.9% of vulnerable or frail patients underwent passive segmentectomy, which might not cause any difference in clinical background, such as age or comorbidities, between the lobectomy and segmentectomy groups. However, further prospective studies are warranted to accurately match the patient background data of the segmentectomy and lobectomy groups. Second, this study excluded 88 patients (20.3%) with missing CT data during the POY3. Third, could not determine the mechanism by which differences in VFA changes affected the non–lung cancer‐related death in this study. Finally, the mechanism by which segmentectomy produces VFA changes was not analysed. Further studies are required to assess the molecular relationship between lung volume reduction and VFA reduction.

In conclusion, we demonstrated that the VFA and WC were better maintained after segmentectomy than after lobectomy. In the lobectomy group, the VFA and WC decreased from POY0.5, and it recovered over a long period. However, in the segmentectomy group, they did not decrease within the third postoperative year. Segmentectomy was associated with a lower likelihood of adipopenia progression.

## Ethics Statement

This study was reviewed and approved by the Institutional Review Board of the Kanagawa Cancer Center (2022 Eki‐54) and followed the tenets of the Declaration of Helsinki. Written‐informed consent was obtained from all the patients. The authors of this manuscript certify that they comply with the ethical guidelines for authorship and publishing in the Journal of Cachexia, Sarcopenia and Muscle [40].

## Conflicts of Interest

The authors declare no conflicts of interest.

## Supporting information


**Figure S1** Measurements of VFA and WC..VFA, visceral fat area; WC, waist circumference.

**Figure S2** Histogram of VFA and WC changes (%) at POY0.5 and POY3..POY, postoperative year; VFA, visceral fat area; WC, waist circumference.

**Figure S3** Comparison of VFA and WC changes in the segmentectomy and lobectomy groups in patients with ≥65 and <65 years of age..a)b) VFA and WC considerably decreased in the lobectomy group within POY3 compared with the segmentectomy group among the patients with ≥65 years of age. c)d) VFA and WC changes between lobectomy and segmentectomy group were similar among the patients with <65 years of age..ANOVA, analysis of variance; POY, postoperative year; VFA, visceral fat area; WC, waist circumference.

**Figure S4** Correlation between the cutoff point of VFA or WC changes at POY3 and HR of OS..HR, hazard ratio; OS, overall survival; POY, postoperative year; VFA, visceral fat area; WC, waist circumference.

**Table S1** Patient characteristics in the PSM lobectomy and segmentectomy groups.
