# Supplementary figures and images for: Comparison of the Changes in Visceral Adipose Tissue After Lobectomy and Segmentectomy for Patients With Early‐Stage Lung Cancer
Source: J Cachexia Sarcopenia Muscle. 2025 Mar 4;16(2):e13751. doi: 10.1002/jcsm.13751 (PMC11876859; doi:10.1002/jcsm.13751)

## Slide 1
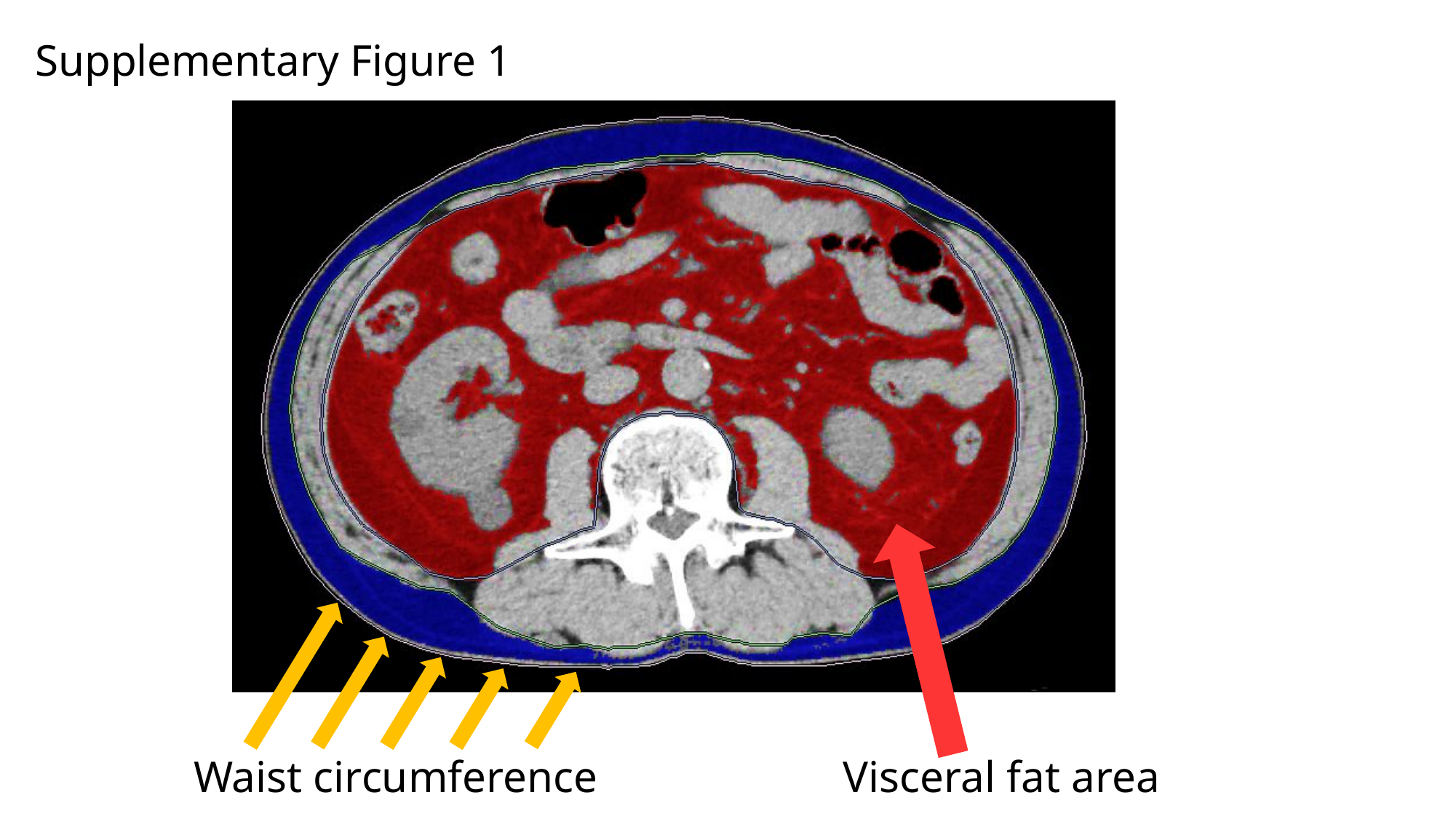

Supplementary Figure 1
Waist circumference
Visceral fat area

Supplement: Supplementary file 1 — Figure S1 Measurements of VFA and WC..VFA, visceral fat area; WC, waist circumference. [file JCSM-16-e13751-s001.pptx]
