# Supplementary material for: Comparison of the Changes in Visceral Adipose Tissue After Lobectomy and Segmentectomy for Patients With Early‐Stage Lung Cancer
Source: J Cachexia Sarcopenia Muscle. 2025 Mar 4;16(2):e13751. doi: 10.1002/jcsm.13751 (PMC11876859; doi:10.1002/jcsm.13751)
Supplement: Supplementary file 2 — Figure S2 Histogram of VFA and WC changes (%) at POY0.5 and POY3..POY, postoperative year; VFA, visceral fat area; WC, waist circumference. [file JCSM-16-e13751-s003.pptx]

## Slide 1
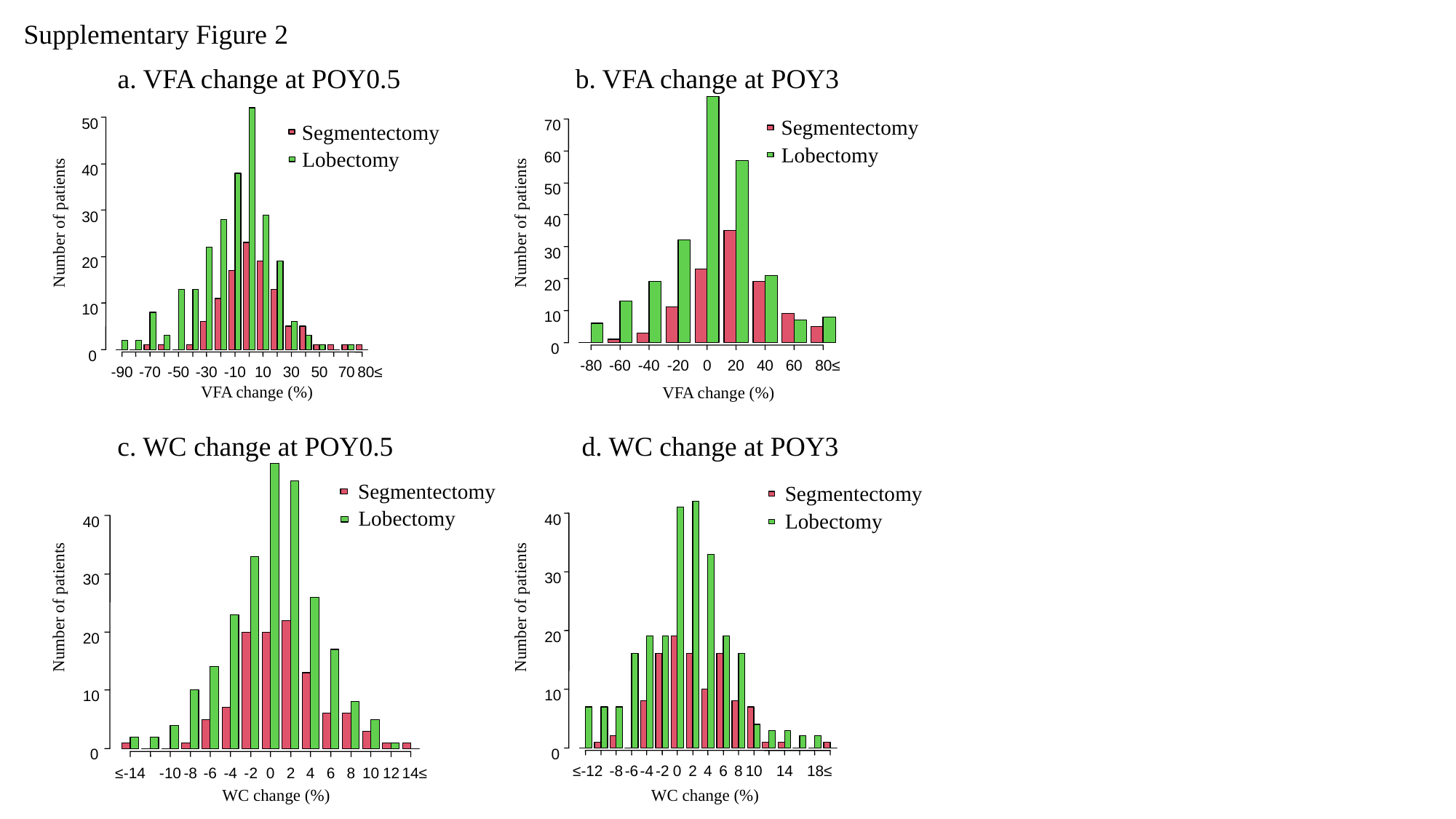

Supplementary Figure 2
b. VFA change at POY3
a. VFA change at POY0.5
Number of patients
Number of patients
70
60
50
40
30
20
10
0
-80
-60
-40
-20
0
20
40
60
80≤
50
40
30
20
10
0
-90
-70
-50
-30
-10
10
30
50
70
Segmentectomy
Segmentectomy
Lobectomy
Lobectomy
80≤
VFA change (%)
VFA change (%)
d. WC change at POY3
c. WC change at POY0.5
40
30
20
10
0
≤-14
-10
-8
-6
-4
-2
0
2
4
6
8
10
12
14≤
Segmentectomy
Number of patients
Number of patients
Segmentectomy
40
30
20
10
0
≤-12
-8
-6
-4
-2
0
2
4
6
8
10
14
18≤
Lobectomy
Lobectomy
WC change (%)
WC change (%)
