# Supplementary material for: Comparison of the Changes in Visceral Adipose Tissue After Lobectomy and Segmentectomy for Patients With Early‐Stage Lung Cancer
Source: J Cachexia Sarcopenia Muscle. 2025 Mar 4;16(2):e13751. doi: 10.1002/jcsm.13751 (PMC11876859; doi:10.1002/jcsm.13751)
Supplement: Supplementary file 3 — Figure S3 Comparison of VFA and WC changes in the segmentectomy and lobectomy groups in patients with ≥65 and <65 years of age..a)b) VFA and WC considerably decreased in the lobectomy group within POY3 compared with the segmentectomy group among the patients with ≥65 years of age. c)d) VFA and WC changes between lobectomy and segmentectomy group were similar among the patients with <65 years of age..ANOVA, analysis of variance; POY, postoperative year; VFA, visceral fat area; WC, waist circumference. [file JCSM-16-e13751-s005.pptx]

## Slide 1
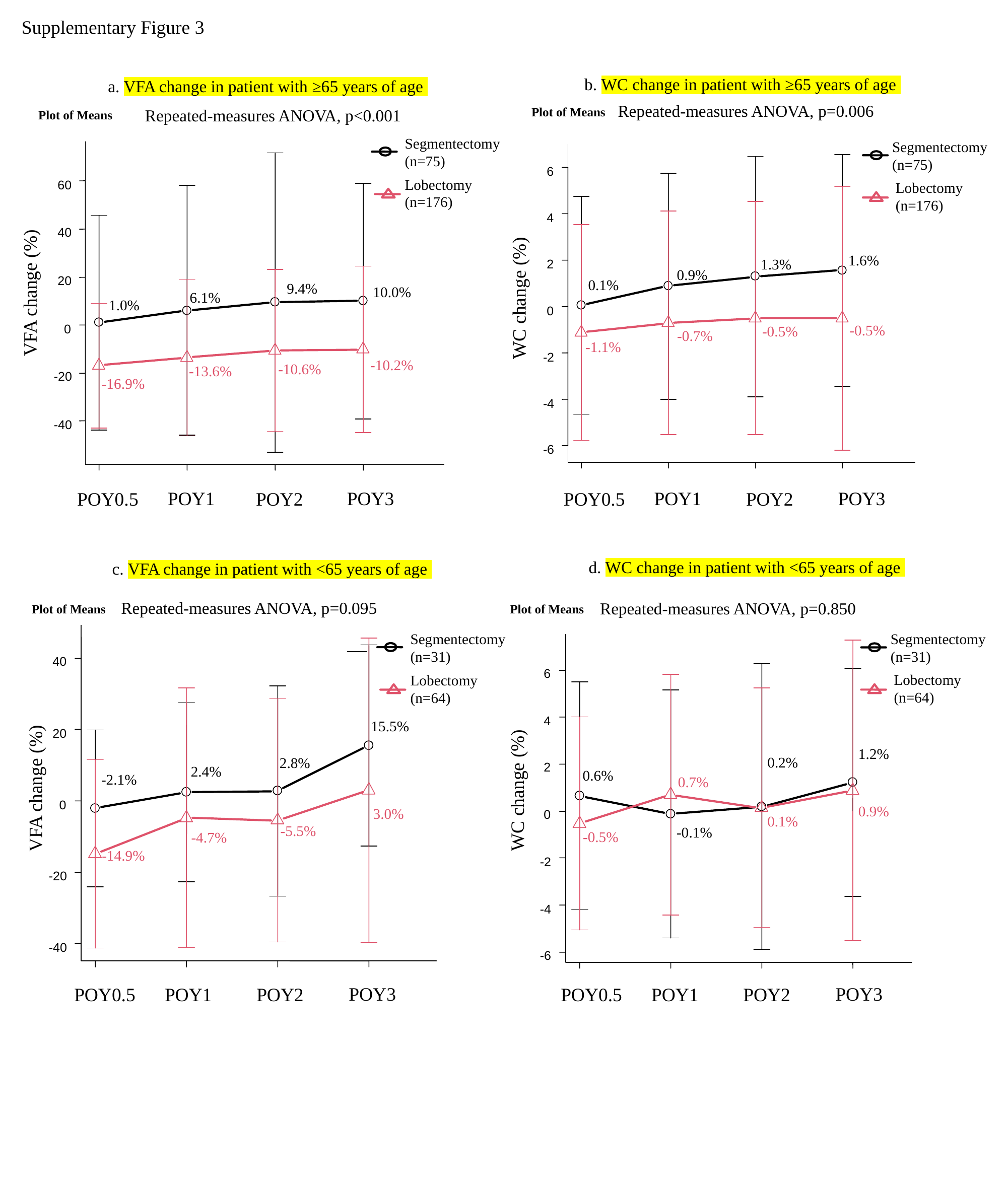

Supplementary Figure 3
b. WC change in patient with ≥65 years of age
a. VFA change in patient with ≥65 years of age
Repeated-measures ANOVA, p=0.006
Repeated-measures ANOVA, p<0.001
Plot of Means
6
4
2
0
-2
-4
-6
Plot of Means
60
40
20
0
-20
-40
Segmentectomy
(n=75)
Segmentectomy
(n=75)
Lobectomy
(n=176)
Lobectomy
(n=176)
VFA change (%)
WC change (%)
1.6%
1.3%
0.9%
0.1%
9.4%
10.0%
6.1%
1.0%
-0.5%
-0.5%
-0.7%
-1.1%
-10.2%
-10.6%
-13.6%
-16.9%
POY3
POY3
POY1
POY1
POY2
POY2
POY0.5
POY0.5
d. WC change in patient with <65 years of age
c. VFA change in patient with <65 years of age
Repeated-measures ANOVA, p=0.095
Repeated-measures ANOVA, p=0.850
Plot of Means
40
20
0
-20
-40
Plot of Means
Segmentectomy
(n=31)
Segmentectomy
(n=31)
6
4
2
0
-2
-4
-6
Lobectomy
(n=64)
Lobectomy
(n=64)
VFA change (%)
WC change (%)
15.5%
1.2%
0.2%
2.8%
2.4%
0.6%
-2.1%
0.7%
0.9%
3.0%
0.1%
-5.5%
-0.1%
-0.5%
-4.7%
-14.9%
POY3
POY3
POY1
POY1
POY2
POY2
POY0.5
POY0.5
