# Supplementary material for: Comparison of the Changes in Visceral Adipose Tissue After Lobectomy and Segmentectomy for Patients With Early‐Stage Lung Cancer
Source: J Cachexia Sarcopenia Muscle. 2025 Mar 4;16(2):e13751. doi: 10.1002/jcsm.13751 (PMC11876859; doi:10.1002/jcsm.13751)
Supplement: Supplementary file 4 — Figure S4 Correlation between the cutoff point of VFA or WC changes at POY3 and HR of OS..HR, hazard ratio; OS, overall survival; POY, postoperative year; VFA, visceral fat area; WC, waist circumference. [file JCSM-16-e13751-s004.pptx]

## Slide 1
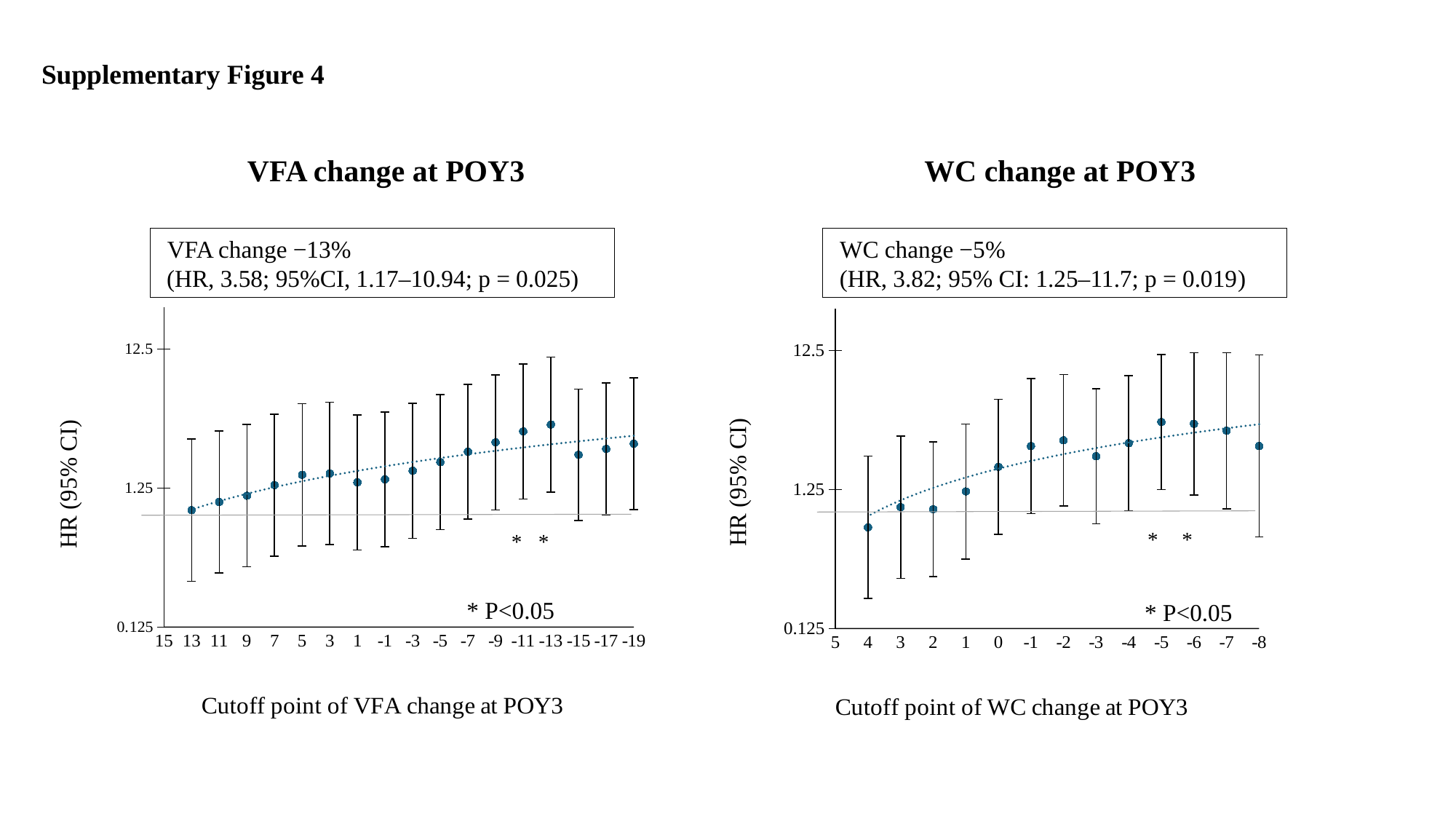

# Supplementary Figure 4
VFA change at POY3
WC change at POY3
 VFA change −13%
 (HR, 3.58; 95%CI, 1.17–10.94; p = 0.025)
 WC change −5%
 (HR, 3.82; 95% CI: 1.25–11.7; p = 0.019)
### Chart
| Category | |
|---|---|
### Chart
| Category | |
|---|---|*
*
*
*
* P<0.05
* P<0.05
