# Supplementary material for: Comparison of the Changes in Visceral Adipose Tissue After Lobectomy and Segmentectomy for Patients With Early‐Stage Lung Cancer
Source: J Cachexia Sarcopenia Muscle. 2025 Mar 4;16(2):e13751. doi: 10.1002/jcsm.13751 (PMC11876859; doi:10.1002/jcsm.13751)
Supplement: Supplementary file 5 — Table S1 Patient characteristics in the PSM lobectomy and segmentectomy groups. [file JCSM-16-e13751-s002.docx]

Supplementary Table. Patient characteristics in the PSM lobectomy and segmentectomy groups

| Total n=186 | Segmentectomy  (n=93) | Lobectomy  (n=93) | P values | SMD |
| --- | --- | --- | --- | --- |
| Age ≥65 | 69 (74.2) | 65 (69.9) | 0.624 | 0.096 |
| Male | 41 (44.1) | 42 (45.2) | 1.000 | 0.022 |
| Body mass index ≤18.5 kg/m^2^ | 8 (8.6) | 4 (4.3) | 0.372 | 0.176 |
| Smoking history | 53 (57.0) | 53 (57.0) | 1.000 | <0.001 |
| Charlson comorbidity index ≥1 | 49 (52.7) | 52 (55.9) | 0.769 | 0.065 |
| Open thoracotomy (vs. cVATS) | 56 (60.2) | 54 (58.1) | 0.881 | 0.044 |
| Adenocarcinoma | 86 (92.5) | 82 (88.2) | 0.458 | 0.146 |
| Tumor size ≥ 2.0cm | 27 (29.0) | 27 (29.0) | 1.000 | <0.001 |
| Pathological stage 0-I (vs. ≥II) | 86 (92.5) | 82 (88.2) | 0.458 | 0.146 |
| Adjuvant chemotherapy | 0 (0) | 0 (0) | 1.000 | <0.001 |

cVATS, complete video-assisted thoracoscopic surgery; PSM, propensity score matching; SMD, standardized mean difference.
